# New Stand for the Compound Microscope

**Published:** 1858-03

**Authors:** O.W. Holmes


*New Stand for the Compound Microscope.* By O. W. Holmes, M. D.

A late number of the Boston Medical Journal contains an account of a new stand for the compound microscope, accompanied by an illustration. We are not familiar with the use of the microscope for any other than anatomical and pathological purposes, and as Dr. Holmes, in addition to his proficiency in this department of microscopic science, is an adept in other matters pertaining to botany, etc., we are hardly able to judge of the extent to which this invention will be useful.

The main object of the invention is to enable the observer to throw direct light upon an object from a lamp under the stage. To accomplish this end the stage is made in the form of a horse-shoe, and the lamp is adjusted by means of some simple and ingenious contrivances which it is unnecessary to describe. Though the invention of Dr. Holmes shows great ingenuity, and accomplishes the ends for which it is intended, we ourselves are unable to see that it will present any practical advantages in anatomical and pathological investigations. According to the experience of the best authorities, the light of day reflected from the sky or a white cloud is the best and purest form of illumination, and next to this is the light of an Aigand lamp, purified, if necessary, by an achromatic condenser. Direct light is seldom, if ever, used. On the whole, the machine presents rather an ungraceful and unpoetical appearance, and as such, we should hardly have looked for it from such a source. We do not think that any one, even the Dr. himself, will be likely to make very frequent use of it.

The instruments of the present day have been brought to such a state of perfection, that one can hardly see any room for a decided innovation; still this may possess advantages of which we are unaware, and the source from which it emanates, bespeaks for it a consideration from practical microscopists.

F.

---

*The American Medical Society in Paris.*—We notice in the February number of the American Medical Monthly, an appeal from the secretary of the American Medical Society in Paris, to the editors of medical periodicals in this country, "to furnish the society, free of expense, the respective journals which they conduct. This call was responded to some time since, but the journals have now been discontinued with the exception of two. It is of the greatest importance that American medical literature should be diffused abroad, and we have in this an admirable opportunity of fulfilling this end. We ourselves, therefore, cheerfully respond to this appeal, and are confident that it will meet with a like reception from all of our editorial confrères. "Gentlemen wishing to accord this favor, will please to forward the Journals to Mr. Paul Bossange, 20 Beekman street, New York, who has generously offered to transmit, free of expense, all books and papers intended for the society. F.

---

*The Hand-book of Practical Receipts for Every-day Use.* A Manual for the Chemist, Druggist, Medical Practitioner and Heads of Families. Comprising the Officinal Medicines, their uses and modes of preparation; and Formulæ for Trade Preparation, Mineral Water, Powders, Beverages, Dietetic Articles, Perfumery, Cosmetics, etc. A Glossary of the Terms used in Chemistry and Medicine, including Old Names, Contractions, Vulgar and Scientific Denomination. With a copious Index to all the Preparations. By THOMAS F. BRANSTON. First American, from the Second London Edition. Philadelphia: Lindsay & Blakiston. 1857.

This little book has met with much favor on the other side of the Atlantic: sufficient, indeed to encourage its re-publication in this country. By reference to the title-page, it will be seen that it contains a number of receipts useful to every one. We can cheerfully recommend it as a very valuable book, and one which is all that it purports to be. F.

---

*Urethro-Vaginal and Vesico-Vaginal Fistula.* Remarks on their Peculiarities and Complications; their Classification and Treatment; Modification of the Button Suture; Report of Cases Successfully Treated. By N. BOZEMAN, M. D., of Montgomery, Ala.

The interesting and original papers which are embodied in this pamphlet have been extracted from the North American Medico-Chirurgical Review for July and Nov., 1857. Dr. Bozeman has acquired quite a reputation within the last few years in the treatment of this distressing and disgusting difficulty. The paper which we have before us is based on the analysis of twenty-five cases, which have been treated by him, and operated upon; in only two of these was the result a failure, and in these he was only partially unsuccessful. We are glad to receive these essays in a distinct form, and in the name of the senior editor, tender our thanks to the author. F.

*Dr. Treadwell's Bequest.*—Mrs. Dorothy, widow of the late Dr. John Dexter Treadwell, and mother of the late Dr. John Treadwell, of Salem, having deceased on the 29th Jan., a large amount of the property left by her son reverts to Harvard College, for the establishment of a professorship of physiology. We have already printed the conditions of the bequest, which are such as, in our opinion, hardly to make it available to the college. We hope that the corporation will decline the trust, in which case it will go to the Massachusetts General Hospital, where it is greatly needed.—*Boston Med. & Surg. Jour., Feb.* 4.

---

*Atlanta Medical College—Resignations and Appointments.*—Professors Boring and Means have resigned their respective Chairs of Obstetrics and Chemistry, in the above institution.

To fill the vacancy caused by the retirement of Professor Boring, Dr. T. S. Powell, of Sparta, has been appointed by the Trustees; we hope that the Institution will be able to secure a successor to Professor Means.

Dr. Means retains his connection as Professor of Chemistry with the Medical College of Georgia.—*Souteern Med. & Surg. Journal.*

---

*Buffalo Medical Association.*—Our present number is deprived of the Proceedings of the Buffalo Medical Association by the adjournment of its regular meeting until Wednesday, Feb. 10th, thus making them too late for this issue. Both the February and March proceedings, therefore, will appear in the next number. F.

---

*Wythes' Pocket Dose Book.*—A very useful and convenient pocket remembrancer of things which are apt to slip from the memory of the practitioner. As such, we know of none which has been more extensively used and is more favorably known to the profession. Published by Lindsay & Blakiston, Philadelphia. F.

